# Understanding psychiatric illness through natural language processing (UNDERPIN): Rationale, design, and methodology

**DOI:** 10.3389/fpsyt.2022.954703

**Published:** 2022-12-01

**Authors:** Taishiro Kishimoto, Hironobu Nakamura, Yoshinobu Kano, Yoko Eguchi, Momoko Kitazawa, Kuo-ching Liang, Koki Kudo, Ayako Sento, Akihiro Takamiya, Toshiro Horigome, Toshihiko Yamasaki, Yuki Sunami, Toshiaki Kikuchi, Kazuki Nakajima, Masayuki Tomita, Shogyoku Bun, Yuki Momota, Kyosuke Sawada, Junichi Murakami, Hidehiko Takahashi, Masaru Mimura

**Affiliations:** ^1^Department of Neuropsychiatry, Keio University School of Medicine, Tokyo, Japan; ^2^Hills Joint Research Laboratory for Future Preventive Medicine and Wellness, Keio University School of Medicine, Tokyo, Japan; ^3^Department of Psychiatry and Behavioral Sciences, Graduate School of Medical and Dental Sciences, Tokyo Medical and Dental University, Tokyo, Japan; ^4^Faculty of Informatics, Shizuoka University, Shizuoka, Japan; ^5^Department of Neuropsychiatry, St. Marianna University School of Medicine Hospital, Kawasaki, Japan; ^6^Computer Vision and Media Lab (Yamasaki Lab), Department of Information and Communication Engineering, Graduate School of Information Science and Technology, The University of Tokyo, Tokyo, Japan; ^7^Keio University School of Medicine, Tokyo, Japan; ^8^Department of Psychiatry, Oizumi Hospital, Tokyo, Japan; ^9^Department of Psychiatry, Koutokukai Sato Hospital, Yamagata, Japan; ^10^Department of Psychiatry, Biwako Hospital, Otsu, Japan

**Keywords:** language, psychiatric disorders, biomarker, machine learning, natural language processing (computer science), neurocognitive disorders

## Abstract

**Introduction:**

Psychiatric disorders are diagnosed through observations of psychiatrists according to diagnostic criteria such as the DSM-5. Such observations, however, are mainly based on each psychiatrist's level of experience and often lack objectivity, potentially leading to disagreements among psychiatrists. In contrast, specific linguistic features can be observed in some psychiatric disorders, such as a loosening of associations in schizophrenia. Some studies explored biomarkers, but biomarkers have yet to be used in clinical practice.

**Aim:**

The purposes of this study are to create a large dataset of Japanese speech data labeled with detailed information on psychiatric disorders and neurocognitive disorders to quantify the linguistic features of those disorders using natural language processing and, finally, to develop objective and easy-to-use biomarkers for diagnosing and assessing the severity of them.

**Methods:**

This study will have a multi-center prospective design. The DSM-5 or ICD-11 criteria for major depressive disorder, bipolar disorder, schizophrenia, and anxiety disorder and for major and minor neurocognitive disorders will be regarded as the inclusion criteria for the psychiatric disorder samples. For the healthy subjects, the absence of a history of psychiatric disorders will be confirmed using the Mini-International Neuropsychiatric Interview (M.I.N.I.). The absence of current cognitive decline will be confirmed using the Mini-Mental State Examination (MMSE). A psychiatrist or psychologist will conduct 30-to-60-min interviews with each participant; these interviews will include free conversation, picture-description task, and story-telling task, all of which will be recorded using a microphone headset. In addition, the severity of disorders will be assessed using clinical rating scales. Data will be collected from each participant at least twice during the study period and up to a maximum of five times at an interval of at least one month.

**Discussion:**

This study is unique in its large sample size and the novelty of its method, and has potential for applications in many fields. We have some challenges regarding inter-rater reliability and the linguistic peculiarities of Japanese. As of September 2022, we have collected a total of >1000 records from >400 participants. To the best of our knowledge, this data sample is one of the largest in this field.

**Clinical Trial Registration:**

Identifier: UMIN000032141.

## Introduction

Psychiatric disorders have a large impact on humans and can reduce quality of life (QOL) because of a high incidence and long duration of illness (1–3). According to global burden of disease surveys conducted by the World Health Organization (WHO) and other organizations, psychiatric disorders, including depression, bipolar disorder, anxiety disorder, schizophrenia, and drug addiction, are the leading medical disorders in terms of years lived with disability (YLDs), accounting for 18.7% of the global YLD in 2019 (4).

Psychiatrists diagnose psychiatric disorders by conducting one-to-one conversations with each patient. Historically, heuristic studies such as linguistics and psychopathology have vigorously studied the thought and language of psychiatric disorders. Several linguistic features are known, such as a loosening of associations in schizophrenia, flight of ideas in mania, psychomotor inhibition in depression, and circumstantial and word recall disorder in dementia. Although there is a common understanding of these linguistic features among psychiatrists, the diagnosis and evaluation of the degree to which a patient deviates from the range of what is considered healthy depend heavily on each psychiatrist's sensitivity and experience. One of the reasons why evaluations remain subjective is that there is no means of quantifying psychiatric diseases to date. These subjective judgments can lead to various problems, such as diagnostic disagreements among psychiatrists, unclear criteria for initiating treatment, and difficulty providing a standardized education of resident doctors. Although the characteristics of the disease appear in each patient's words, it is sometimes difficult to diagnose atypical cases or cases with very severe symptoms, as it can be difficult to determine what symptoms are causing the difficulty in coherent speech.

The absence of objective methods to detect psychiatric diseases has prompted several research to investigate biomarkers. Biomarkers were often obtained from some large datasets of EEG (electroencephalography) (5–7), fMRI (functional magnetic resonance imaging) (8–10), genomes (11, 12). Furthermore, using data-driven methods, these datasets aid in the discovery of novel biotypes and the extraction of cross-diagnostic characteristics (13, 14). These have the ability to forecast the diagnosis and course of an illness as well as the creation of novel therapies. In addition to EEG, fMRI and genomes, linguistic and acoustic features can also be used for the detection of biomarkers.

In recent years, there has been an increase in the number of reported studies that have attempted to diagnose psychiatric disorders using natural language processing (NLP). Target disorders include schizophrenia (15–20), depression (21), bipolar affective disorder (22), obsessive-compulsive disorder (23), autism spectrum disorders (24), dementia (25, 26) and many others. Many studies have utilized a wide range of NLP techniques from the morphological and syntactic analysis (27) to novel approaches for representing textual information such as n-grams (28), word2Vec (29), ELMo (Embedding from Language Models) (30), and BERT (Bidirectional Encoder Representations from Transformers) (31). Some studies have supposed the unique models that could explain specific pathological features such as poverty of thoughts (32), loosening of associations (33), thought disorder in psychosis (34), etc. A few studies have comprehensively analyzed speech data using and comparing the techniques reported in previous studies (35, 36). For example, Corcoran et al. have utilized the part-of-speech tagging analyses (which measures syntactic complexity) and Latent Semantic Analysis (which measures semantic coherence) to verify the machine learning classifiers based on these NLP methods for predicting psychosis onset (35). Morgan et al. have compared the abilities of the 12 NLP measures to differentiate speech data from subjects at clinical high risk for psychosis, first-episode psychosis patients, and healthy controls (36). Those measures included the total number of words, the number of ambiguous pronouns, the semantic coherence using Latent Semantic Analysis, and some indicators calculated by the speech graphs [developed by Mota et al. (34)]. Although findings of very accurate illness onset prediction have been published (37), there are still many open challengers: the lack of large datasets, the extraction of numerous features that are straightforward and reliable for clinical application, the search for features that are applicable to a variety of diseases, and analyses of the correlation between the disease time course and disease severity.

The present study aims to create large datasets of the Japanese language with a great number of samples, types, and amount of utterances and look into ways to extract linguistic and audio indicators that can be used to differentiate between healthy-disease and disease-disease. Such datasets are limited at present (38). They could enable us to obtain reliable results and bring pioneering insights from NLP in the Japanese-speaking region. Notably, this study aims to target 300–500 participants and obtain 600–2,500 corpora; as such, it will be one of the largest participants' records among studies examining the diagnosis of major psychiatric disorders using NLP. This research also aims to deepen our understanding of the characteristics of psychiatric disorders and quantify them using NLP and machine learning so that the features of psychiatric disorders can be identified and quantified in the future. Such technology could eventually be used for early diagnosis and prevention.

## Methods

### Dataset creation

In this section, we are going to describe the methods to create large Japanese-language datasets of adult patients diagnosed with major psychiatric disorders (depression, bipolar disorder, schizophrenia, anxiety disorder (including obsessive-compulsive disorder), mild cognitive impairment, or dementia) and healthy controls. We do not plan to make the datasets openly available because the possibility of containing personal information cannot be ruled out. They will be available to our collaborators and researchers who use them for scientific purposes after completing the ethics application modification process.

#### Participants

This study is a multicenter, prospective, observational study. Subject recruitment was started in 2018 and is ongoing. Participants are being recruited at seven hospitals and three outpatient clinics in Japan. Patient recruitment is being conducted at the following locations and hospitals: Tokyo (Keio University Hospital, Tsurugaoka Garden Hospital, Oizumi Hospital, Komagino Hospital, Oizumi Mental Clinic, Asakadai Mental Clinic), Kanagawa (Nagatsuta Ikoinomori Clinic), Shiga (Biwako Hospital), Yamagata (Sato Hospital), and Fukushima (Asaka Hospital). Participants are inpatients or outpatients aged ≥20 years who meet the diagnostic criteria for depression, bipolar disorder, schizophrenia, anxiety disorder (including obsessive-compulsive disorder), mild cognitive impairment, or dementia according to the DSM-5 or ICD-11. Healthy volunteers will consist of healthy individuals with no history of psychiatric disorders who have volunteered to participate in the study after reading the research group's website and printed recruitment advertisements and who are at least 20 years old at the time of consent. Researchers will obtain written informed consent about participation in this study and data storage from all the participants. In cases where the patients are judged to be decisionally impaired, the patients' guardians will be asked to provide consent. Participants will be able to leave the study at any time. This study was approved by the Institutional Review Board of Keio University School of Medicine and the participating medical facilities.

#### Data collection

The following data will be collected in this study. We will assign the study number to each participant and manage the data. The demographic characteristics, the conversation data (the voice data and the text information), and the clinical assessment results will be linked to the study number and separated from the identifiers such as name, address, and so on. They will be strictly stored in the server locked by password considering the possibility that the voice data might include identifiers such as names.

##### Demographic characteristics

Sex, age, diagnosis, diagnosis subtype, duration of illness, prescription, and complications will be collected from the medical records. We will also collect as much information as possible about their education, birthplace, outpatient or inpatient status, years of education, educational background, occupation, work status, employment status, income, marital status, number of years of married (or divorced or separated), number of children, number of people living together, etc. If some information is missing, the patients will be asked to provide the information.

##### Conversation data

Up to a 60-min conversation with a participating psychiatrist or psychologist will be conducted. We will confirm that the participant speaks Japanese-language as the primary. A participating psychiatrist or psychologist will avoid including the participant's identifiers in the recorded part of the conversation as much as possible. The conversation consists of three parts: free conversation, picture-description task, and story-telling task. During free conversation, a participant speaks with a psychiatrist or psychologist about the course of the illness, the patient's current condition, daily life, sleep patterns, interests and hobbies, and comments on news, movies, stories, and pictures, etc. No strict structuring is performed. During picture-description task, participants are presented with three pictures: one depicting three children eating donuts used in Visual Perception Test for Agnosia (VPTA), one showing the waterside used in COGNISTAT and one representing the shore used in WAB. The patients are asked to explain them in as much detail as possible. Pictures are presented in the same order to all participants. In story-telling task, participants are asked to describe the story of Cinderella in as much detail as possible. For example, a psychologist or a psychiatrist asks them, “Please think about how you would explain the story to someone who does not know the story.” If the participant does not know the story of Cinderella, the famous Japanese folktale “Kaguya-Hime” is used instead. The picture-description task and story-telling task each take about 10 min. In all the conversations, an interviewer is required to ask questions using neutral words, not using emotional-valenced words, to make an interjection, and to dig into the participant's story. The interviewer and participant are asked to wear microphone headsets named PRO8HE (39) (its range of the frequency of sampling is from 200 to 18,000 Hz, and its sensitivity is −55dB.), and the conversation is audio-recorded in a consultation room or similar quiet environment.

##### Clinical assessment

The severity of the illness will be assessed using the rating scales described in the table (Table 1). In addition, the treating psychiatrists will be asked to evaluate each patient's current disease severity using the Clinical General Impression Scale-Severity (CGI-S). A structured psychiatric diagnostic interview (SCID-IV-TR) will be conducted to the greatest degree feasible to confirm the diagnoses during the follow-up interview.

**Table 1:** Data collection and visit schedule.

			**Number of visits**				
			**1**	**2**	**3**	**4**	**5**
			**Assessments must be done in Visit 1 and Visit 2**		**Assessments will be done as long as possible**		
A) Demographic characteristics	All participants	Demographic characteristics	✓				
		Information on treatment history	✓	✓	✓	✓	✓
	Only patients	SCID-IV-TR	✓ (Once during research period)				
B) Conversation with a psychiatrist or psychologist	All participants	free conversation, picture-description task, and story-telling task	✓	✓	✓	✓	✓
C) Severity assessment using clinical rating scales	C-1) Monopolar depression disorder or Bipolar disorder or Anxiety disorder	HAM-D MADRS YMRS HAM-A STAI SWLS Cantril's Ladder of life scale FS-J Subjective Well-being Inventory	✓	✓	✓	✓	✓
	C-2) Schizophrenia	PANSS SWLS Cantril's Ladder of life scale FS-J Subjective Well-being Inventory	✓	✓	✓	✓	✓
	C-3) Neurocognitive disorder	MMSE CDR MoCA-J WMS-R LM SWLS Cantril's Ladder of life scale FS-J Philadelphia Geriatric Center Morale Scale Subjective Well-being Inventory	✓	✓	✓	✓	✓
	For healthy volunteers	*MMSE (*: Performed before all of the other tests)	✓				
		All of C-1), C-2), C-3)	✓	✓	✓	✓	✓

FS-J, The Japanese version of the Flourishing Scale; SWLS, Satisfaction With Life Scale, SCID, Structural Clinical Interview for DSM-5; HAM-D, Hamilton Depression Rating Scale; MADRS, Montgomery-Asberg Depression Rating Scale; YMRS, Young Mania Rating Scale; STAI, State-Trait Anxiety Inventory; PANSS, Positive And Negative Syndrome Scale; MMSE, Mini-Mental State Examination; MoCA-J, Montreal Cognitive Assessment-Japanese version; WMS-R LM, Wechsler Memory Scale-Revised Logical Memory; CDR, Clinical Dementia Rating; M.I.N.I., Mini-International Neuropsychiatric Interview.

Healthy volunteers will be screened using the Mini-International Neuropsychiatric Interview (M.I.N.I.) and the Mini-Mental State Examination (MMSE). They will be excluded if they have a history of psychiatric disorders or cognitive impairment. For the M.I.N.I., if any diagnosis is found, and for the MMSE, if the score is below 27, the participant will not be allowed to participate in the study as a healthy subject.

In this study, not only the collection of linguistic information associated with each disease but also the relationship between illness severity and linguistic information will be important. Therefore, we will follow the participants two to five times during the study period to interview the subjects at times when the severities of their symptoms differ (i.e., severe, moderate, mild, and during remittance). An interval of at least 1 month between the two assessments will be required. The same procedures described above will be conducted for the follow-up data collection.

#### Data processing and annotation

The acquired voice data will be converted into text information using speech recognition technology developed by the research group and will be compensated manually; annotations, such as pauses, fillers, nods, interjections, repetitions, co-supplementation, lexical responses, incomplete sentences, misstatements, etc., will also be made. We will mask the part of personal identifiers such as names when converting voice data into text information. At the same time, the following phonic information will be extracted from the recorded audio data to utilize the physical properties of the audio data in the machine learning model: fundamental frequency (F0); first, second, and third formant frequencies (F1, F2, F3); cepstral peak prominence (CPP); and Mel-frequency cepstrum coefficients (MFCC). These are the major features in speech-language analysis (40). Software such as Praat (41) and openSMILE (42) will be used to extract such phonic information.

Based on the annotated text data thus obtained, various variables are calculated using NLP techniques, such as morphological and syntactic analyses using MeCab (43, 44) and JUMAN (45, 46). For instance, these analyses will include the frequency of the occurrence of each part of speech, vocabulary (negative and positive words), syntactic complexity, length of the utterance, frequency of occurrence of person, use of pronouns and distance to the referent, use of case structure (“te, ni, wo, ha”), and so on. We will use NLP techniques to represent textual data as n-grams and word embeddings obtained from pre-trained models such as BERT (47).

Linguistic features considered to be characteristic of specific psychiatric diseases will be selected and statistically compared between groups (e.g., patients vs. healthy subjects, patients in different disease groups). This step is necessary to verify whether each variable has pathological validity before using it for machine learning.

### Machine learning

The machine learning models in this study will be trained to perform the following tasks: (1) to predict whether a subject has or does not have psychiatric disorders such as depression, bipolar disorder, anxiety disorder, schizophrenia, or dementia; (2) to predict the severity of a subject's state based on the results of rating scales such as the Hamilton Rating Scale for Depression (HAM-D), the Young Mania Rating Scale (YMRS) for bipolar disorder, the Positive And Negative Syndrome Scale (PANSS) for schizophrenia, and MMSE for dementia; (3) to predict the improvement or deterioration of a subject's disorder with respect to a previously recorded state if the subject has undergone a prior assessment; and 4) to predict the scores of individual items in clinical rating scales that are indicators of different aspects of a subject's disorder, such as positive symptoms, negative symptoms, and disorganized symptoms for schizophrenia. The data used to train these machine learning models may include voice features extracted from voice data obtained from subjects, conversational and linguistic information from transcribed text data, and annotations assigned to the data described above.

Next, we will need to perform feature engineering and design the architecture of machine learning. The data, represented through NLP techniques, together with the features obtained through feature engineering processes, will be then used to extract disease features. In particular, disease features extraction will be performed relying on machine learning models such as decision trees, support vector machines (SVM), and deep learning architectures. Each model will be evaluated using cross-validation and then the relative importance of each feature will be estimated using a feature importance estimation method such as XGBoost. We will then evaluate the results using the mean absolute error, coefficient of determination, and correlation coefficients. We also plan to verify whether the features obtained in this manner can distinguish firstly between a normal and disease state, secondly among different diseases, and whether they can distinguish changes in disease severity over time.

### Sample size

The results of a pilot study conducted on 30 participants prior to the main study showed that the combined NLP and machine learning had an accuracy of 72–80% for binary classification to differentiate between disease and healthy controls (based on a leave-one-out cross-validation). The demographic data in the pilot study are as follows. Age (years, mean ± SD): 56.8 ± 16.9, Gender (%male): 46.7, Diagnosis: 5 schizophrenia, 1 bipolar disorder, 4 depression, 5 anxiety disorder, 5 neurocognitive disorder, and 10 healthy controls.

Specifically, we used the part of speech information as well as feature vectors extracted from the data points using an NLP package called SpaCy as the input to train an XGBoost model for predicting the labels of the data points. We cross-validated the XGBoost model using leave-one-out cross-validation (LOOCV) where we would leave one data point as the test data and train an XGBoost model using the remaining data points. The process repeated for each data point and computed the accuracy of the LOOCV as the ratio of correctly predicted data points out of all of the data points. We further optimized the hyperparameters of the XGBoost model by choosing the set of parameters that has the highest LOOCV accuracy.

We constructed a learning curve to estimate the number of samples required to achieve a classification accuracy of 90%. Starting from a sample size of 15, we randomly sample 15 data points from the total of 30 data points and perform the LOOCV as described above, and repeat the random sampling of 15 data points 10 times, each time keeping track the optimal classification accuracy to get an idea of the distribution of the accuracy for 15 data points.

We then repeat the whole process at one-sample increments until we reach 29 samples. The resulting accuracies are then fitted to a linear model. Based on the parameters of the fitted linear model, we estimated the number of data points required to achieve an accuracy of 90%.

We calculated that a minimum of 50 samples would be required. In addition, considering the variability of the data over a wide range of age groups, severity of illness, and regional differences, it was estimated that about 10 times the number of samples would be necessary. Another means of approximating sample sizes for regression analysis is that the number of cases should be approximately 10 times the number of the independent variables used to obtain statistical confidence (48).

In the pilot study, we used 62-dimensional features for speech information and linguistic information. These features include linguistic features such as the number of morphemes, the number of vocabulary, the number of nouns, verbs, and adjectives, the number of particles “ga” etc., and the number of conjunction, and non-linguistic features such as the total time, the response time, the number of fillers, the formants. In the present study, we plan to use a total of about 100 independent variables for the analysis. We will also be employing dimension reduction and feature selection techniques to reduce the number of independent variables in the model. Based on the approximations, we estimate that the desired sample size is approximately 1,000. It should also be noted that the actual needed number of samples is likely to be lower.

## Discussion

The UNDERPIN study is unique in its purpose, the novelty of its methods, such as the use of NLP, and the size of the data sample. It has a broad and evolving perspective that is expected to shed light on traditional psychiatry by applying the new analytical method of machine learning to language. In addition, the large and Japanese-language datasets enhance the novelty. As of September 2022, we have collected a total of >1,000 datasets from >400 participants (102 patients diagnosed with schizophrenia, 89 with depression, 58 with bipolar disorder, 35 with anxiety disorder, and 79 with neurocognitive disorder). The large datasets to be created in this study have the potential of leading to the development of new biomarkers.

Psychiatrists have long relied on language and superficial behavioral data to diagnose psychiatric disorders. Although many empirical studies have explored biomarkers of mental illness (49, 50), no robust biomarkers have been identified that are clinically practicable.

This study will focus on the linguistic features of psychiatric disorders, which have been explored using traditional disciplines such as linguistics (51, 52) and psychopathology. By introducing the method of NLP, we will be able to bring an empirical and mathematical perspective to humanistic examinations. Quantifying linguistic features will allow a more detailed examination of their correlation with other objective measures, such as brain imaging studies. Boer et al. identified features that distinguish patients with schizophrenia and healthy subjects based on speech data; using the calculated features, they then predicted the integrity of the white matter language tracts in two groups (53). Although such studies remain rare (54), they may bring clinical practice and research on psychiatric disorders, which have been suggested to be divergent, closer together.

Moreover, the results of this study could lead to early detection and early intervention for patients with psychiatric disorders. As the concept of duration of untreated psychosis (DUP) suggests (55), early detection and intervention are essential for a favorable disease prognosis.

Furthermore, we plan to apply the findings of this research to text data collected from social networking services, such as Twitter. We expect that this would enable us to analyze larger-scale data longitudinally and to infer the mood of society and its relationships among social events. For this purpose, we will also construct our own Japanese sentiment polarity dictionary to conduct a detailed analysis.

We might extract cross-lingual and cross-cultural pathological features, comparing the findings from these Japanese-language datasets with those from other linguistic areas such as the English-speaking region. Two studies have concerned the cross-lingual pathological features using NLP. They examined the cross-lingual generalizability focusing on incoherent speech (56) and vocal abnormalities (57). Each generalizability was not high as a result. They pointed out that it is necessary to ensure a sufficient sample size in each linguistic area and take the heterogeneity of the sample population into account. Our large datasets might provide more robust findings in the Japanese-speaking region.

The challenges of this research are as follows: the difficulty of keeping inter-rater reliability high, the scarcity of previous or similar studies, findings, and datasets in the Japanese-speaking region, and the peculiarity of the Japanese language. First, it will be essential to ensure a similar annotation protocol among the examiners. Similarly, it will be very important to keep the inter-rater reliability high when diagnosing and/or assessing patients. Anticipating this issue, the study team has developed educational modules to maintain a high quality of ratings, and the inter-rater reliability will be tested using random sampling during the study period. Second, as far as we know, this is the first study of this kind to be conducted on such a large sample size in a Japanese-speaking region. The quantity of publicly available conversational data in the Japanese language is relatively small, and almost none is from speakers with mental disorders. To date, most of the reported cases of NLP applied to psychiatric disorders have been conducted in English-speaking areas; therefore, it might be difficult to replicate the findings of the previous studies directly in this study. Finally, we discuss the linguistic characteristics of the Japanese language. Some features cannot exist in Japanese due to differences in grammar. For example, the determiners used to analyze linguistic features of psychosis (37) do not exist in Japanese. Secondly, the Japanese language is agglutinative in that it does not use spaces between words, so its word boundaries are not as clear as they are in other languages. Another challenge involves handling spoken conversational data, which tends to be broken or ill-formed. Furthermore, the Japanese language has the characteristic of often omitting arguments such as subjects. At the same time, however, if common features capable of identifying specific psychiatric disorders across languages can be found, such information would likely become an important disease feature. Although there are many challenges, this research will enable us to study the languages of psychiatric disorders statistically and comprehensively, and it will provide a new interpretation of language to psychiatrists.

## Preprint and previous presentation

This article has been posted on the preprint site: https://doi.org/10.1101/2021.12.05.21267037.

The design of the study and the dataset that was acquired were briefly presented at the International Workshop on Health Intelligence, 2019 (58).

## Ethics statement

The studies involving human participants were reviewed and approved by the Institutional Review Board of Keio University School of Medicine and the participating medical facilities. The patients/participants provided their written informed consent to participate in this study.

## Author contributions

TKis, HN, YK, K-cL, and TY wrote the manuscript. YE, MK, KK, AS, AT, TH, YK, TKik, KN, MT, SB, YM, KS, JM, HT, and MM critically reviewed the manuscript. All authors participated in the design of the study. All authors contributed to the article and approved the submitted version.

## Funding

This research was supported by the Japan Science and Technology Agency CREST under Grant Number JPMJCR1684 and JPMJCR19F4. The 1st grant was awarded in 2016 and ended in 2018, the 2nd grant was awarded in 2019 and ended in 2021 and the 3rd grant was awarded in 2022 and will end in 2024. The funding source did not participate in the design of this study and will not be involved in the study's execution, analyses, or submission of results.

## Conflict of interest

TKis has received consultant fees from FRONTEO. YE has received speaker's honoraria from Eisai. TH received speaker's honoraria from Yoishi-tomi. TKik has received speaker's honoraria from Astellas, Dai-nippon Sumitomo, Eli Lilly, Janssen, MSD, Otsuka, Yoshitomi Yakuhin, Pfizer, and Takeda. JM has received speaker's honoraria from Eli Lilly, Janssen, Otsuka, MSD, Shionogi, and Pfizer. MM has received speaker's honoraria from Daiichi Sankyo, Dainippon-Sumitomo Pharma, Eisai, Eli Lilly, Fuji Film RI Pharma, Janssen Pharmaceutical, Mochida Pharmaceutical, MSD, Nippon Chemipher, Novartis Pharma, Ono Yakuhin, Otsuka Pharmaceutical, Pfizer, Takeda Yakuhin, Tsumura, and Yoshitomi Yakuhin. Also, MM received grants from Daiichi Sankyo, Eisai, Pfizer, Shionogi, Takeda, Tanabe Mitsubishi, and Tsumura. The remaining authors declare that the research was conducted in the absence of any commercial or financial relationships that could be construed as a potential conflict of interest.

## Publisher's note

All claims expressed in this article are solely those of the authors and do not necessarily represent those of their affiliated organizations, or those of the publisher, the editors and the reviewers. Any product that may be evaluated in this article, or claim that may be made by its manufacturer, is not guaranteed or endorsed by the publisher.

## Abbreviations

UNDERPIN: Understanding Psychiatric Illness Through Natural Language Processing
DSM-5: Diagnostic and Statistical Manual of Mental Disorders, Fifth Edition
ICD-11: International Classification of Diseases 11th Revision
UMIN: University Hospital Medical Information Network
M.I.N.I.: Mini-International Neuropsychiatric Interview
MMSE: Mini-Mental State Examination; Mini-International Neuropsychiatric Interview
QOL: Quality Of Life
YLDs: years lived with disability
NLP: natural language processing
ELMo: Embeddings from Language Models
BERT: Bidirectional Encoder Representations from Transformers
CGI-S: Clinical General Impression Scale-Severity
SCID: Structural Clinical Interview for DSM-5
HAM-D: Hamilton Depression Rating Scale
MADRS: Montgomery Asberg Depression Rating Scale
YMRS: Young Mania Rating Scale
STAI: State-Trait Anxiety Inventory
PANSS: Positive And Negative Syndrome Scale
MMSE: Mini-Mental State Examination
MoCA-J: Montreal Cognitive Assessment-Japanese version
WMS-RLM: Wechsler Memory Scale-Revised Logical Memory
CDR: Clinical Dementia Rating
F0: fundamental Frequency
F1: 1st formant Frequency
F2: 2nd formant Frequency
FS-J: The Japanese version of the Flourishing Scale
SWLS: Satisfaction With Life Scale
F3: 3rd formant Frequency
CPP: Cepstral Peak Prominence
MFCC: Mel-Frequency Cepstrum Coefficients
SVM: support vector machines
LOOCV: leave-one-out cross-validation
DUP: duration of untreated psychosis.
